# Reduced auditory evoked gamma-band response and schizophrenia-like clinical symptoms under subanesthetic ketamine

**DOI:** 10.1038/s41386-019-0328-5

**Published:** 2019-02-06

**Authors:** Stjepan Curic, Gregor Leicht, Stephanie Thiebes, Christina Andreou, Nenad Polomac, Iris-Carola Eichler, Lars Eichler, Christian Zöllner, Jürgen Gallinat, Saskia Steinmann, Christoph Mulert

**Affiliations:** 1 Psychiatry Neuroimaging Branch, Department of Psychiatry and Psychotherapy, University Medical Center Hamburg-Eppendorf, Hamburg, Germany; 2 Institute for Sex Research and Forensic Psychiatry, Center of Psychosocial Medicine, University Medical Center Hamburg-Eppendorf, Hamburg, Germany; 3 Center for Psychotic Disorders, University Psychiatric Hospital, University of Basel, Basel, Switzerland; 4 Department of Anesthesiology, University Medical Center Hamburg-Eppendorf, Hamburg, Germany; 5 Department of Psychiatry and Psychotherapy, University Medical Center Hamburg-Eppendorf, Hamburg, Germany; 6 Centre for Psychiatry and Psychotherapy, Justus Liebig University, Gießen, Germany

**Keywords:** Schizophrenia, Neurophysiology

## Abstract

Abnormal gamma-band oscillations (GBO) have been frequently associated with the pathophysiology of schizophrenia. GBO are modulated by glutamate, a neurotransmitter, which is continuously discussed to shape the complex symptom spectrum in schizophrenia. The current study examined the effects of ketamine, a glutamate N-methyl-d-aspartate receptor (NMDAR) antagonist, on the auditory-evoked gamma-band response (aeGBR) and psychopathological outcomes in healthy volunteers to investigate neuronal mechanisms of psychotic behavior. In a placebo-controlled, randomized crossover design, the aeGBR power, phase-locking factor (PLF) during a choice reaction task, the Positive and Negative Syndrome Scale (PANSS) and the Altered State of Consciousness (5D-ASC) Rating Scale were assessed in 25 healthy subjects. Ketamine was applied in a subanaesthetic dose. Low-resolution brain electromagnetic tomography was used for EEG source localization. Significant reductions of the aeGBR power and PLF were identified under ketamine administration compared to placebo (*p* < 0.01). Source-space analysis of aeGBR generators revealed significantly reduced current source density (CSD) within the anterior cingulate cortex during ketamine administration. Ketamine induced an increase in all PANSS (*p* < 0.001) as well as 5D-ASC scores (*p* < 0.01) and increased response times (*p* < 0.001) and error rates (*p* < 0.01). Only negative symptoms were significantly associated with an aeGBR power decrease (*p* = 0.033) as revealed by multiple linear regression. These findings argue for a substantial role of the glutamate system in the mediation of dysfunctional gamma band responses and negative symptomatology of schizophrenia and are compatible with the NMDAR hypofunction hypothesis of schizophrenia.

## Introduction

Cognition, perception, and consciousness require synchronized gamma-band oscillations (GBO) [1]. Abnormal GBO might be responsible for dysfunctional neural circuitry causing perceptual and cognitive disturbances in schizophrenia [2, 3]. Among sensory GBO in schizophrenia, auditory [4, 5], and visually evoked gamma-band responses [2] have been explored most frequently. Evoked sensory gamma-band responses represent both basic sensory processing, as well as higher cognitive functions like attention and memory, by which they seem to be influenced [6]. The auditory-evoked gamma-band response (aeGBR) has been observed in the timeframe of 25–100 ms after a stimulus and has been shown to be sensitive to task-related cognitive demands [7, 8]. A reduction of the aeGBR power and degree of phase locking over trials has been shown across all stages of schizophrenia: in first episode patients [5], chronic patients [9], subjects with a high-risk of developing psychosis [10], as well as symptom-free first-degree relatives of patients with schizophrenia [11].

The interplay between fast-spiking parvalbumin (PV)-expressing gamma-aminobutyric acid (GABA-) releasing interneurons (PV^+^-interneurons), and glutamatergic pyramidal cells is essential in the generation of gamma-frequency rhythmicity [12]. On different levels, presynaptic and postsynaptic aberrations in PV^+^-interneurons are supposed to limit adaptability of GBO in the prefrontal cortex (PFC) along with reduced cognitive control [13]. Particularly, the glutamate N-methyl-d-aspartate receptors (NMDAR) on the PV^+^-interneurons are thought to be essential in the generation of GBO [14]. Moreover, there is evidence that the function of genes associated with schizophrenia is related to the NMDAR hypofunction [15]. Due to this, alterations of GBO have been proposed as an intermediate phenotype of schizophrenia [11] with a putative application in the early detection of the disease.

The NMDAR-antagonists ketamine and phencyclidine (PCP) are known to induce psychopathological changes similar to those seen in schizophrenia with respect to positive, negative, and cognitive symptoms [16]. This led to the assumption that the involvement of the NMDAR could play an important role in the pathogenesis of schizophrenia [17]. The conceptualization of the glutamate hypothesis of schizophrenia was reinforced by observations in the ketamine model of schizophrenia on different levels in both humans and animal models, as well as basic scientific research involving the NMDAR [18].

Typical and atypical antipsychotic agents have been the main pharmacological treatment option in schizophrenia for decades. By targeting the dopamine D2-receptors, they alleviate positive symptoms but have less impact on negative symptoms, which are the main reason for poorer functional outcomes [19]. Several lines of evidence, arising from the ketamine model of schizophrenia, indicate that negative symptoms could be treated targeting the glutamatergic system. The degree of ketamine-induced negative symptoms, not positive symptoms, is correlated with the occupancy of the NMDAR by ketamine [20]. Several, but not all studies using NMDAR-modulating substances like glycine, d-serine, and sarcosine have shown to reduce negative symptoms [21]. Thus, there is a need for neurophysiological markers, like the aeGBR, that predict response and outcome of glutamatergic interventions in schizophrenia. In an animal model of schizophrenia, Nakamura et al. revealed a correlation between phase-locked gamma oscillations, PV^+^-interneurons and negative-symptom-like behavioral deficits, suggesting phase-locked gamma oscillations as a biomarker in humans for measuring the effectiveness of pharmacological interventions [22].

The aeGBR sources are located within bilateral auditory cortices and in frontal midline structures of the brain including the dACC, however, only the dACC-source is elevated in cognitively demanding tasks in healthy subjects [8]. In patients suffering predominantly negative symptoms, GBO in the ACC increases with task-difficulty, while the opposite effect was observed for patients with dominant positive symptoms [23]. Previous studies with ketamine have been investigating oscillatory responses with passive tasks without cognitive demands like paired clicks or the auditory steady-state response [24, 25]. Although the aeGBR is one of the best established neurophysiological markers for schizophrenia and the relationship between GBO and NMDAR-mediated glutamatergic neurotransmission is remarkable, the aeGBR has not been explored in a paradigm sensitive to task-related cognitive demands under ketamine administration in healthy volunteers so far.

Based on the strong relationship between glutamatergic neurotransmission, the NMDAR function, the GBO as well as negative symptoms, the present study aims to establish a translational link between evidence from preclinical studies and from studies investigating patients with schizophrenia. We hypothesized (1) a ketamine-induced schizophrenia-like reduction of the aeGBR power and degree of phase locking accompanied by (2) the occurrence of schizophrenia-like symptoms and task-related behavioral changes (increased reaction times and error rates) as well as (3) a specific association between the ketamine-induced aeGBR alterations and the severity of ketamine-induced negative symptoms. Furthermore, based on previous EEG und MEG studies [7, 8], we expected a specific decrease in the generator of the aeGBR in the anterior cingulate cortex (ACC).

## Participants and methods

### Participants

Twenty-eight healthy male subjects were enrolled in this study. The general procedure was approved by the Ethics Committee of the Medical Association Hamburg. Written informed consent was obtained according to the latest version of the Declaration of Helsinki. Two participants withdrew consent shortly before the first session, one of the participants dropped out due to adverse effects (dissociative effect/headache). For the following data analysis, 25 subjects with a mean age of 25 years (SD = 2.64) were included. The sample of the study is part of a larger project, of which some results have been published [26, 27].

Participants were recruited from the community through advertisement. All volunteers were right-handed, assessed with the Edinburgh Handedness Inventory (mean = 82.20, SD = 16.89). Language comprehension and verbal IQ (mean = 112.96, SD = 5.95) were tested with the German WST vocabulary test. Normal hearing was tested by audiometry as previously described [28].

Exclusion criteria were left-handedness, the use of illicit drugs, acute or past psychiatric disorders—tested with the Mini International Neuropsychiatric Interview (M.I.N.I.) and the Schizotypal Personality Questionnaire (SPQ)—and health conditions that represented a contraindication to the administration of ketamine.

### Psychometric assessment

Psychiatric symptomatology was assessed using the Positive and Negative Syndrome Scale (PANSS). Prior to the first intervention (baseline) and after both drug challenges (placebo/ketamine) psychopathological evaluation was conducted by an experienced psychiatrist. PANSS scores were evaluated using the five-factor model by van der Gaag et al. [29].

The Altered State of Consciousness (5D-ASC) questionnaire [30], a visual analog scale, was used after both drug challenges (ketamine/placebo) to assess the subjective effects of ketamine. This self-rating questionnaire consists of 94 items assessing key dimensions of altered states of consciousness.

### Study design

We used a placebo-controlled, randomized cross-over study design. The subjects were not informed as to treatment-condition and treatment condition was not confirmed by study personnel. During the ketamine session, a subanesthetic dose of *S*-ketamine hydrochloride (Ketanest^®^ S—Pfizer) was administered intravenously in 0.9% sodium chloride (NaCl) solution for a total length of 75 min. The ketamine infusion was started with an initial bolus of 10 mg over 5 min followed by a maintenance infusion of 0.006 mg/kg/min. The time between sessions was at least 7 days. As ketamine plasma levels slowly increase with continuous infusion [31], the dosage was reduced by 10% every 10 min. Placebo was administered analogously as 0.9% NaCl infusion. Heart rate, blood pressure, oxygen saturation, and vigilance of the subjects were continuously monitored during both sessions. The clinical raters were not blinded with respect to condition due to the obvious clinical effects of ketamine.

### Paradigms

We used an auditory reaction task [32] that has been shown earlier to increase the aeGBR amplitude according to the level of difficulty [7]. One hundred and twenty tones (duration: 250 ms, generated using the Presentation software version 16.1) of different pitches (33% 800 Hz, 33% 1000 Hz, and 33% 1200 Hz) were presented via earphones at about 85 dB SPL with pseudo-randomized interstimulus intervals (ISI: 2.5–7.5 s; mean = 5.0 s). Subjects were asked to respond differentially by pressing a button with the left index finger following the low tone (800 Hz) and with the right index finger following the high tone (1200 Hz). No response was required for the middle (1000 Hz) tone. Prior to each run, subjects were instructed to respond as fast and as accurately as possible. Reaction times (from stimulus onset until button press) and errors (incorrect response or no response within 2000 ms after stimulus presentation) were registered during the experimental run.

### EEG recording and pre-processing

In a sound-attenuated and electrically shielded room, the subjects were seated with their eyes open in a slightly reclined chair with a head rest and were asked to look at a fixation cross presented at a 19‶ computer monitor. The EEG was recorded with a sampling rate of 1000 Hz, with 64 active electrodes mounted on an elastic cap (ActiCaps, Brain Products, Munich, Germany) using the Brain Vision Recorder software Version 1.10 (Brain Products, Munich, Germany). Electrodes were positioned in an extended 10/20 system with the additional positions: AF7, AF3, AF4, AF8, F5, F1, F2, F6, F10, FT9, FT7, FC3, FC4, FT8, FT10, C5, C1, C2, C6, TP7, CPz, TP8, P5, P1, P2, P6, PO3, POz, and PO4. Eye movements were recorded through four EOG channels. An electrode at the FCz position was used as the reference, the electrode at position AFz served as ground. Impedances were always kept below 5 kΩ.

Data analysis was carried out using Brain Vision Analyzer (BVA) Version 2.1.0.327 (Brain Products, Munich, Germany). The channels PO9 and PO10 were excluded from further analysis due to persistent muscle artifact contamination in most subjects. After band-pass filtering (1–100 Hz), topographic interpolation (spherical splines) of up to three channels was performed (mean number of interpolated channels: placebo condition 0.08 ± 0.4; ketamine condition 0.19 ± 0.63; no significant differences between conditions). Channels were selected for interpolation if more than 5% of data in the respective channel was affected by technical artifacts, or muscle artifacts exceeding amplitudes of ±70 µV. The continuous EEG was segmented into epochs of 1400 ms starting 400 ms prior to the auditory stimulus. Segments including incorrect responses or amplitudes exceeding ±70 µV within a 410 ms window starting 210 ms pre-stimulus in any channel were automatically rejected. Saccadic spike potential artifacts (SPs) in the gamma frequency range [33] were controlled by an additional “radial electro-oculogram channel” (REOG), that was derived following the procedure described by Keren [34]. Independent component analysis (ICA) was applied to identify and remove blinks, drifts, muscle artifacts, and SPs based on their characteristic topographies, time courses, and frequency distributions [35] . After re-referencing to common average reference and baseline correction (using an interval of 210–10 ms prestimulus), averaged event-related potential (ERP) waveforms were computed. Only waveforms based on at least 35 segments were accepted.

### aeGBR power and phase-locking factor (PLF)

Using the BVA Software, the aeGBR power and PLF were computed using a wavelet transformation [complex Morlet wavelet with the formula *w*(*t*) = *A* exp(−*t*²/2)exp(*i*2*πct*), Morlet parameter *c* = 5, instantaneous amplitude (Gabor) normalization] that has been used in several previous studies [36].

In order to reveal the phase-locked evoked gamma power, wavelet transformation was performed on averaged ERP. Layer-wise baseline correction was applied using a baseline of 200 ms starting 210 ms prior to stimulus presentation. The frequency range from 20 to 80 Hz was divided into 30 frequency steps (distributed on a logarithmic scale). For aeGBR peak detection, in accordance with previous studies [5, 8, 10, 11], the wavelet layer with the central frequency of 40 Hz (frequency range 32–48 Hz) was extracted. Based on previous studies [7, 9, 36] the aeGBR-peak was defined as the highest value within the timeframe 30–100 ms poststimulus at the electrode Cz.

The PLF was calculated by performing an additional wavelet transformation (without layer-wise baseline correction) at the single trial level and extracting complex-phase information with all vector lengths normalized to the unit circle (“Phase locking factor” and “Complex Data Measures” solution, BVA software) before averaging of the phase information. Gamma PLF peaks were defined as the highest value of the wavelet layer centered around 40 Hz within the timeframe 30–100 ms poststimulus at the electrode Cz.

### Source analysis of aeGBR

Source-space localization analyses were performed with the low-resolution brain electromagnetic tomography (LORETA) KEY software package (http://www.uzh.ch/keyinst/loreta) [37]. The EEG source localization of the aeGBR across 35–45 Hz was executed for every subject within a time window from 30 to 70 ms post stimulus.

Region-of-interest (ROI) analyses were performed for the dACC (dACC-ROI), the primary (BA-41-ROI) as well as the secondary (BA-42-ROI) auditory cortices. The dACC-ROI consisted of all voxels within 10 mm radius of the previously identified maximum activity in the dACC during the auditory choice reaction task [9]. For the BA-41-ROI and BA-42-ROI predefined regions from the LORETA software package ROI-maker for BA-41 and BA-42 were chosen.

The voxel-wise comparison of cortical activities between conditions was done using a one-tailed *t*-test (statistical significance threshold *p* < 0.05) for paired groups implemented in the sLORETA software. A statistical nonparametric mapping randomization method was used to automatically adjust for multiple comparisons with a Fisher’s random permutation test with 5000 randomizations.

### Statistical analyses

All other statistical analyses were performed using IBM SPSS Statistics Version 22. Comparisons of 5D-ASC scores, aeGBR power and PLF, ROI-current source density (CSD), as well as reaction times and error rates between placebo and ketamine condition, were done using paired-sample *t*-tests. PANSS scores were analyzed by one-way repeated measure analysis of variance (RM-ANOVA) with session (baseline, placebo, and ketamine) as within-subject factors. Moreover, we conducted a multiple linear regression analysis with relative aeGBR-decrease (compared to placebo) as the dependent variable and the occurrence of schizophrenia-like symptoms in the ketamine condition (five PANSS factors) as predictors. All five PANSS factors were simultaneously entered into the model. In all analyses, the significance level was set to *α* = 0.05. Bonferroni corrections were applied to adjust for multiple comparisons.

## Results

### Ketamine-induced behavioral changes

**Fig. 1:**
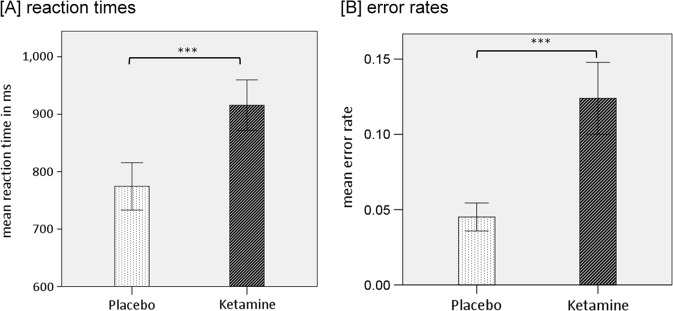
Bar charts of the mean values of reactions times (**a**) and error rates (**b**) with error bars representing ±1 standard errors of the mean (***p < 0.001)

Ketamine significantly increased reaction times (mean = 916 ms, SD = 211) compared to the placebo condition (mean = 774 ms, SD = 198; *t*(22) = −3.78, *p* < 0.001). In addition, participants showed significantly increased error rates in the ketamine condition (mean = 12.4%, SD = 11.5%) compared to the placebo condition (mean = 4.5%, SD = 4.5%); *t*(22) = −3.4, *p* < 0.001) (Fig. 1).

### Ketamine-induced psychopathological symptoms

**Fig. 2:**
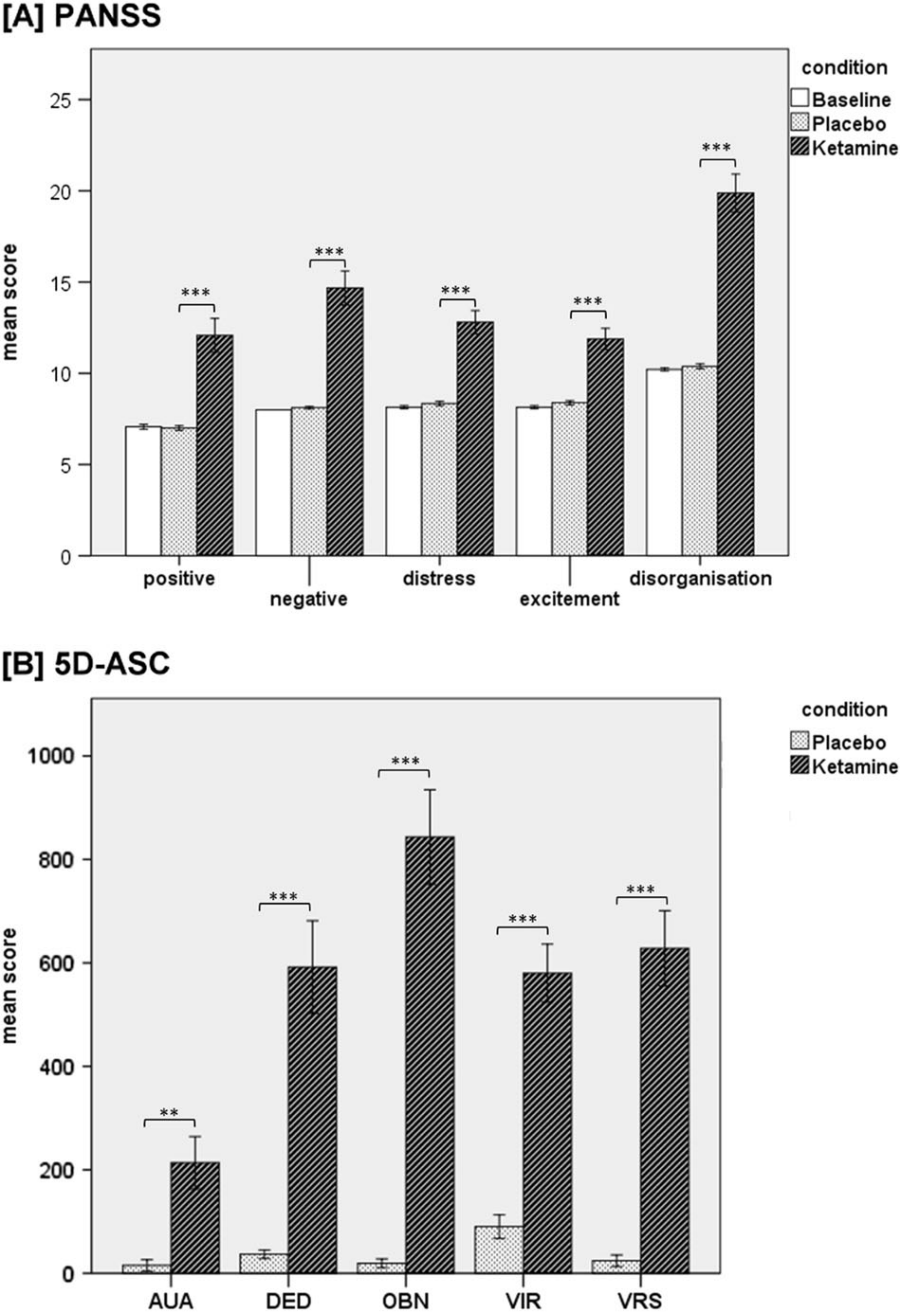
Bar charts of the mean values of the five PANSS subscores (**a**) and the 5D-ASC-scale total and subscores (**b**) with error bars representing ±1 standard errors of the mean, Bonferroni-corrected for multiple comparisons (***p < 0.001, **p < 0.01). AUA auditory alterations, DED dread of ego dissolution, OBN oceanic boundlessness, VIR vigilance reduction, VRS visionary restructuralization

The RM-ANOVA showed significant effects of session on all PANSS scores and subscores (PANSS total: *F*(2,76) = 117.15, *p* < 0.001; Positive: *F*(2,76) = 30.49, *p* < 0.001; Negative: *F*(2,76) = 54.18, *p* < 0.001; Disorganization: *F*(2,76) = 90.18, *p* < 0.001; Excitement: *F*(2,76) = 38.80, *p* < 0.001; Distress: *F*(2,76) = 51.74, *p* < 0.001). Post-hoc *t*-tests showed that the PANSS total score and all subscores were significantly increased after ketamine administration compared to placebo administration and baseline values. There were no significant differences with respect to any PANSS score between baseline and placebo condition (Fig. 2a).

Ketamine led to a statistically significant increase in all of the 5D-ASC scores compared to placebo condition (Fig. 2b). There was a significant difference for the auditory alterations subscale between placebo (*M* = 15.3, SD = 54.5) and ketamine (*M* = 213.6, SD = 253.6; *t*(24) = −3.736, *p* < 0.01), for the dread of ego dissolution subscale between placebo (*M* = 36.4 SD = 41.8) and ketamine (*M* = 592.0, SD = 447.4; *t*(24) = −6.371, *p* < 0.001), for the oceanic boundlessness subscale between placebo (*M* = 18.3, SD = 18.1) and ketamine (*M* = 843.4, SD = 456.4; *t*(24) = −8.915, *p* < 0.001), for the vigilance reduction subscale between placebo (*M* = 89.6, SD = 114.3) and ketamine (*M* = 580.2, SD = 280.6; *t*(24) = −7.573, *p* < 0.001) and for the visionary restructuralization subscale between placebo (*M* = 25.2, SD = 58.1) and ketamine (*M* = 621.2, SD = 370.6; *t*(24) = −7.665, *p* < 0.001).

### Ketamine-induced changes of aeGBR and PLF

**Fig. 3:**
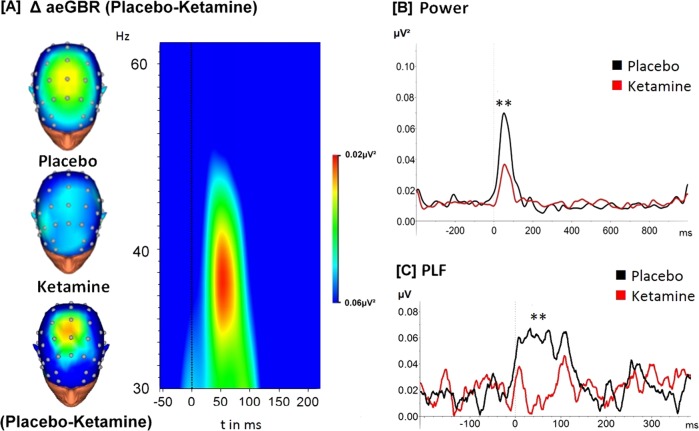
**a** Time–frequency analysis of the mean difference (placebo-ketamine) of the auditory evoked gamma-band response (aeGBR) power in the timeframe between 50 ms pre-stimulus and 200 ms post-stimulus. The aeGBR can be seen as an increased activity about 50 ms after stimulus presentation (dashed line) and around 40 Hz in the frequency range. Scalp topographies are displayed for each condition as well as a difference topography in the same scaling as the time–frequency analysis. The scalp topographies were calculated for the timeframe 50–75 ms and a frequency of 40 Hz. The aeGBR power (**b**) and phase-locking factor (PLF) (**c**) are displayed as the results of the wavelet analysis (complex Morlet wavelet) focused on the activity around 40 Hz for the ketamine condition (red line) and the placebo condition (black line) (**p < 0.01)

Around 50 ms after stimulus presentation, an increase of the evoked gamma activity compared to the baseline level was observed at electrode Cz in both conditions. In the ketamine condition the aeGBR power was significantly decreased (mean = 0.04, SD = 0.05) compared to the placebo condition (mean = 0.11, SD = 0.12); *t*(24) = 2.90, *p* < 0.01 (Fig. 3a, b). Regarding the PLF, participants receiving ketamine showed significantly decreased PLF peaks (14 mean = 0.21; SD = 0.10) compared to the placebo condition (mean = 0.27; SD = 0.14); *t*(24) = 2.49, *p* < 0.01 (Fig. 3d). Elevated resting state gamma-oscillations are consistently observed following ketamine [38], which also applies for the baseline CSD in this study (Fig. S2B). In order to examine a putative relationship between ketamine-induced elevated resting state gamma activity and reduced aeGBR, we performed a correlation analysis between these measures, which yielded no significant result (see supplementary material, Fig. S2). The N100 amplitude, representing lower frequencies, was reduced under ketamine (Fig. S3), but did not correlate with aeGBR power or PFL.

### LORETA ROI analysis

**Fig. 4:**
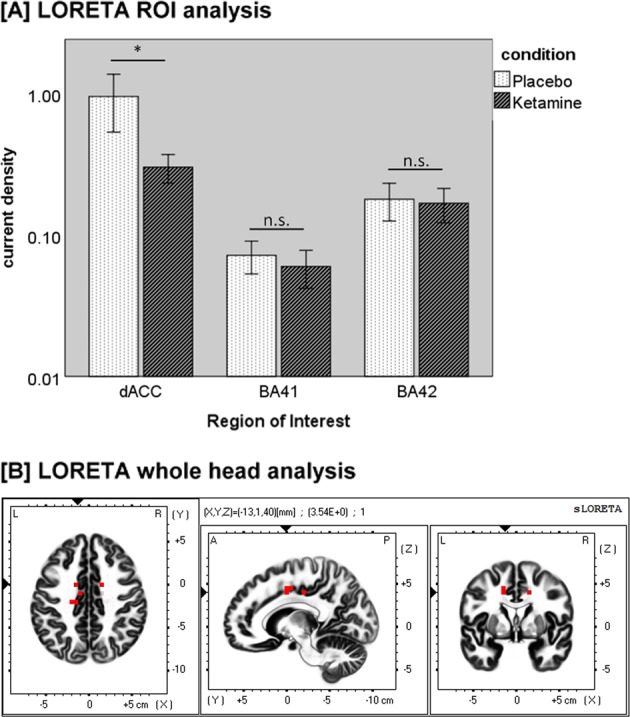
**a** Mean current source density (µA/m²) in the dACC-ROI, BA-41-ROI, and BA-42-ROI with error bars representing ±1 standard errors of the mean (*p < 0.05; n.s. = not significant). **b** Difference map of low-resolution brain electromagnetic tomography (LORETA) source activity in the gamma-frequency band (35–45 Hz) comparing the current source density (CSD) of the aeGBR between the placebo and ketamine conditions. The red voxels represent areas of significant voxel-wise differences after correction for multiple comparisons (*p* < 0.05). Red voxels show a reduced CSD in the dorsal anterior cingulate cortex (dACC) under ketamine

A paired-samples *t*-test was conducted to compare the CSD in the three ROIs between placebo and ketamine condition (Fig. 4a). While there was a significant difference between placebo (*M* = 0.80, SD = 1.45) and ketamine (*M* = 0.30, SD = 0.34) condition for the dACC-ROI (*t*(23) = 1.743, *p* = 0.047), there was no significant difference for the BA41-ROI (Placebo: *M* = 0.07, SD = 0.10; Ketamine: *M* = 0.06, SD = 0.08; *t*(23) = 0.543, *p* = 0.296) or the BA42-ROI (Placebo: *M* = 0.18, SD = 0.27; Ketamine: *M* = 0.18, SD = 0.24; *t*(23) = 0.117, *p* = 0.454).

### LORETA whole head analysis

The comparison of the aeGBR across 35–45 Hz revealed only significantly reduced cortical source activities under ketamine in the dorsal anterior cingulate cortex (dACC) (Brodmann area 24), with an asymmetry in favor of the left hemisphere after correction for multiple comparisons; *t* > 3.350, *p* < 0.05 (Fig. 4b).

### Association between neuropsychological and psychopathological variables

**Fig. 5:**
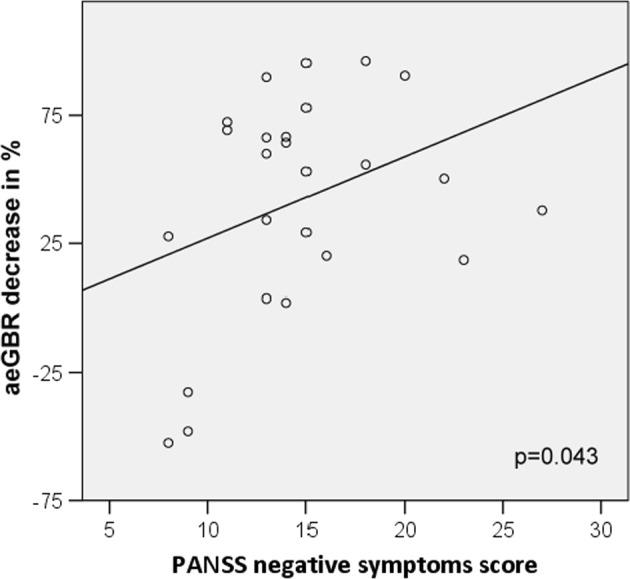
Positive correlation between the relative decrease of aeGBR power and the Positive and Negative Syndrome Scale (PANSS) negative factor (Pearson’s *r* = 0.342)

The multiple linear regression analysis indicated that only the PANSS negative score under ketamine influence significantly predicted the relative decrease of the aeGBR power (*F*^1,19^ = 5.304; *p* = 0.033), while other factors were not significantly associated with the aeGBR power decrease (all *p* > 0.27; see supplementary material), when controlling for the effect of the other symptoms. There was also a positive correlation between the PANSS negative score and the relative decrease of the aeGBR power in the post-hoc bivariate correlation analysis (Pearson’s *r* = 0.352; *n* = 25; *p* = 0.042) (Fig. 5); the correlations of the other factors are shown in Fig. S1 (all *p* ≥ 0.34).

## Discussion

Using a placebo-controlled, randomized, pharmacological 64-channel-EEG crossover study design, we showed for the first time that ketamine reduces the ability to increase the aeGBR power, PLF and its generator within the dorsal ACC in response to a cognitively demanding auditory choice reaction task in healthy subjects. This result is in line with our hypothesis of a ketamine-induced schizophrenia-like reduction of the aeGBR due to its NMDAR inhibiting effect, which has been suggested to successfully model the proposed dysfunction of the glutamatergic system as a core pathophysiological mechanism of schizophrenia. Ketamine also caused behavioral changes similar to those seen in patients with schizophrenia. Namely, schizophrenia-like psychopathological symptoms as assessed by PANSS score and 5D-ASC score, as well as reaction times and error rates, were significantly increased in the ketamine condition compared to placebo. We observed a significant association between the decrease of aeGBR power in the ketamine condition and the degree of ketamine-induced schizophrenia-like negative symptoms both when controlling for the effect of the other symptoms and in the bivariate post-hoc analysis.

High-frequency oscillations in the gamma-frequency range are known to be altered in schizophrenia patients [39]. The aeGBR has been shown to be reduced across all stages of schizophrenia [5, 9–11]. AeGBR sources within the auditory cortices and the dorsal ACC as revealed by EEG [7] and MEG source localization [8] as well as simultaneous measurement of EEG and fMRI [36] have been reported to be less active in patients with schizophrenia [9] and subjects at high risk of psychosis [10]. Reduced glutamate concentration, e.g. in the ACC of schizophrenia patients [40] may be one reason for a reduced aeGBR generation in schizophrenia. The generation of GBO in the human brain critically involves a neural microcircuit consisting of glutamatergic pyramidal cells and fast-spiking inhibitory GABA-releasing PV^+^-interneurons [12].

This microcircuit shows several abnormalities in patients with schizophrenia [41]. Thus, alterations of GBO in schizophrenia patients have been interpreted as a correlate of a disturbed glutamatergic neurotransmission in schizophrenia [15]. Supporting these findings, we found a reduction of the aeGBR induced by the NMDAR antagonist ketamine.

Accordingly, a ketamine-induced reduction of gamma activity has been shown in animal studies [42, 43]. Therefore, the results of the present study support the idea of the ketamine challenge as a model of glutamatergic disturbances underlying schizophrenia and provide a link between preclinical studies and observations in schizophrenia patients. Contrary to our results increased gamma activity during the application of ketamine in humans has been reported [24, 25]. However, the effect shown for the auditory steady-state response (ASSR) in the gamma frequency range seems to depend on the duration and dose of the ketamine administration: A recent animal study reported temporally dynamic patterns of ketamine-induced gamma changes with a negative correlation between NMDAR occupancy by ketamine and the ASSR phase stability over trials at a certain time point [44], which indicates a rather dynamic than static effect of ketamine on gamma responses. Most resting state studies suggest an increase of ketamine-induced gamma-band activity as revealed by both MEG [45] and EEG [46] studies, which is in line with results from increased resting state GBO connectivity in schizophrenia patients [47]. While we observed a decrease in aeGBR power and PLF, Thiebes et al. showed increased interhemispheric connectivity in the gamma-band frequency range between bilateral auditory cortices in the same study population [27]. These results resemble complex neurophysiological changes seen in schizophrenia patients.

In the present study a not significant increase of baseline-activity in the gamma-range under ketamine was observed (see supplement information, Fig. S2). However, the strong reduction of the aeGBR power and PLF under ketamine cannot be explained by baseline gamma-band differences (no difference between baseline gamma-band activity between ketamine and placebo at Cz, no correlation between baseline gamma-band activity and the aeGBR). In line with our results, Carlen et al. could both demonstrate an increased resting state GBO, while light-activation of channelrhodopsin-expressing PV+-interneurons failed to evoke higher GBO, in an animal model [14]. In this context, the aberrant function of PV+-interneurons may explain the co-occurrence of both elevated resting state GBO and reduced aeGBR under ketamine. However, the E/I imbalance that has been identified as a fundamental contributing factor in the generation of disturbed GBO [48] may not be due to NMDAR-mediated PV+-interneurons malfunction alone. There is evidence that PV+-interneurons should be less affected by NMDA receptor antagonism [49], which underlines the importance of the NMDA receptors on other neuron population like pyramidal cells [50] and somatostatin (SST) interneurons. Especially SST-interneurons may contribute to defective gamma-oscillations in addition to PV+-interneurons [51].

Our findings of ketamine-induced schizophrenia-like symptoms across all investigated dimensions (positive and negative symptoms, distress, disorganization, and excitement) further validate the proposed overlap between the ketamine model of schizophrenia and the disease itself [52]. Furthermore, this is supported by behavioral changes, prolonged reaction times and increased error rates, in the ketamine condition, which are in line with schizophrenia studies [9, 10]. Moreover, similar results have been observed in other ketamine studies both in humans [53] and animals [54].

The observed association of aeGBR decrease in the ketamine condition and ketamine-induced schizophrenia-like negative symptoms leads to the assumption of an association between aeGBR, negative symptoms and the NMDAR. In contrast, Hong et al. showed a positive correlation between ketamine-induced negative symptoms and the amount of gamma oscillatory activity evoked by auditory paired clicks [24]. Nevertheless, our finding is paralleled by reports on a negative relationship between the ability to generate gamma oscillatory activity and the degree of negative symptoms in schizophrenia patients [5], although other schizophrenia studies did not report such correlations [55]. In this study, an attention-requiring aeGBR-measure was related to negative symptoms. However, preattentive neurophysiological measures of the mismatch negativity (MMN) were also related to negative symptoms in the same study population [26]. There was no statistically significant correlation between the MMN measures from Thiebes et al. [26] and the measures of aeGBR (data available upon request). In conclusion negative symptoms may be the consequence of both attention-requiring and preattentive NMDAR-mediated neurophysiological changes. Our results provide a potential biomarker for negative symptoms underlying glutamatergic deficits and are in line with the findings from Thiebes et al. who showed an association between the MMN and negative symptoms in the same study population [26].

Our source-localization corroborate findings of fMRI-studies, that identified the ACC as the main brain region to be affected by ketamine (review by Haaf et al. [38]). We are aware of the limited spatial resolution of EEG-source-localizations compared to fMRI [56] and hope that further studies using combined pharmacological EEG-fMRI will provide improved source-localization.

A limitation of this study is that ketamine-effects may have led to unblinding of the subjects. Instead of controlling with NaCl solution, future studies could use dopamine-agonists to simultaneously test the glutamate and dopamine hypotheses of schizophrenia and to reduce the likelihood of unblinding. Our study, like others, was limited by a missing validation of the PANSS scale in healthy subjects. However, consistent overlap between PANSS ratings of ketamine-induced symptoms and schizophrenia symptoms, especially for negative symptoms [57], provides a strong argument for PANSS assessment in this study population. Nonetheless further validation of the five-factor model of the PANSS in this study population would support better comparability with schizophrenia patients.

In conclusion, we report a placebo-controlled ketamine-induced reduction of power and phase stability of the aeGBR in a sample of healthy subjects performing a cognitively demanding auditory choice-reaction task. We identified a reduced activation of the aeGBR generator within the dorsal ACC under ketamine. In view of very similar results in schizophrenia patients, the NMDAR antagonizing effect of ketamine, the crucial role of the NMDAR in the generation of GBO, and NMDAR-related alterations in schizophrenia; these results strongly support the glutamate hypothesis of schizophrenia. Moreover, our study establishes a link between preclinical and schizophrenia patients studies, highlighting the ketamine model of schizophrenia as a putative tool for further investigations of glutamatergic treatment options and the identification of a subgroup of schizophrenia patients who benefit from such drugs (reviewed in ref. [58]).

## Supplementary information

Supplemental Material
