# Supplementary material for: Reduced auditory evoked gamma-band response and schizophrenia-like clinical symptoms under subanesthetic ketamine
Source: Neuropsychopharmacology. 2019 Feb 6;44(7):1239–46. doi: 10.1038/s41386-019-0328-5 (PMC6785009; doi:10.1038/s41386-019-0328-5)
Supplement: Supplementary file 1 — Supplemental Material [file 41386_2019_328_MOESM1_ESM.pdf]

## Association between neuropsychological and psychopathological variables

The multiple linear regression analysis with relative decrease of aeGBR power as dependent variable and the 5 factors of the Positive and Negative Syndrome Scale (PANSS) as predictors, revealed only the PANSS negative factor as a significant predictor of aeGBR power decrease (p=0.033), when controlling for the effect of the other symptoms.

In this model the other factors (emotional distress factor [p=0.597], positive symptoms factor [p=0.968], excitement factor [p=0.270] and disorganization factor [p=0.597] ) were no significant predictors for the relative decrease of aeGBR power. For bivariate correlations between PANSS and aeGBR-decrease see Fig. S1.

## Baseline-activity

Resting-state activity is known to be elevated following ketamine-administration in humans [1] and rodents (reviewed in [2]), therefore we tested the baseline-activity in the paradigm and the possible link between elevated baseline-activity and eaGBR measures.

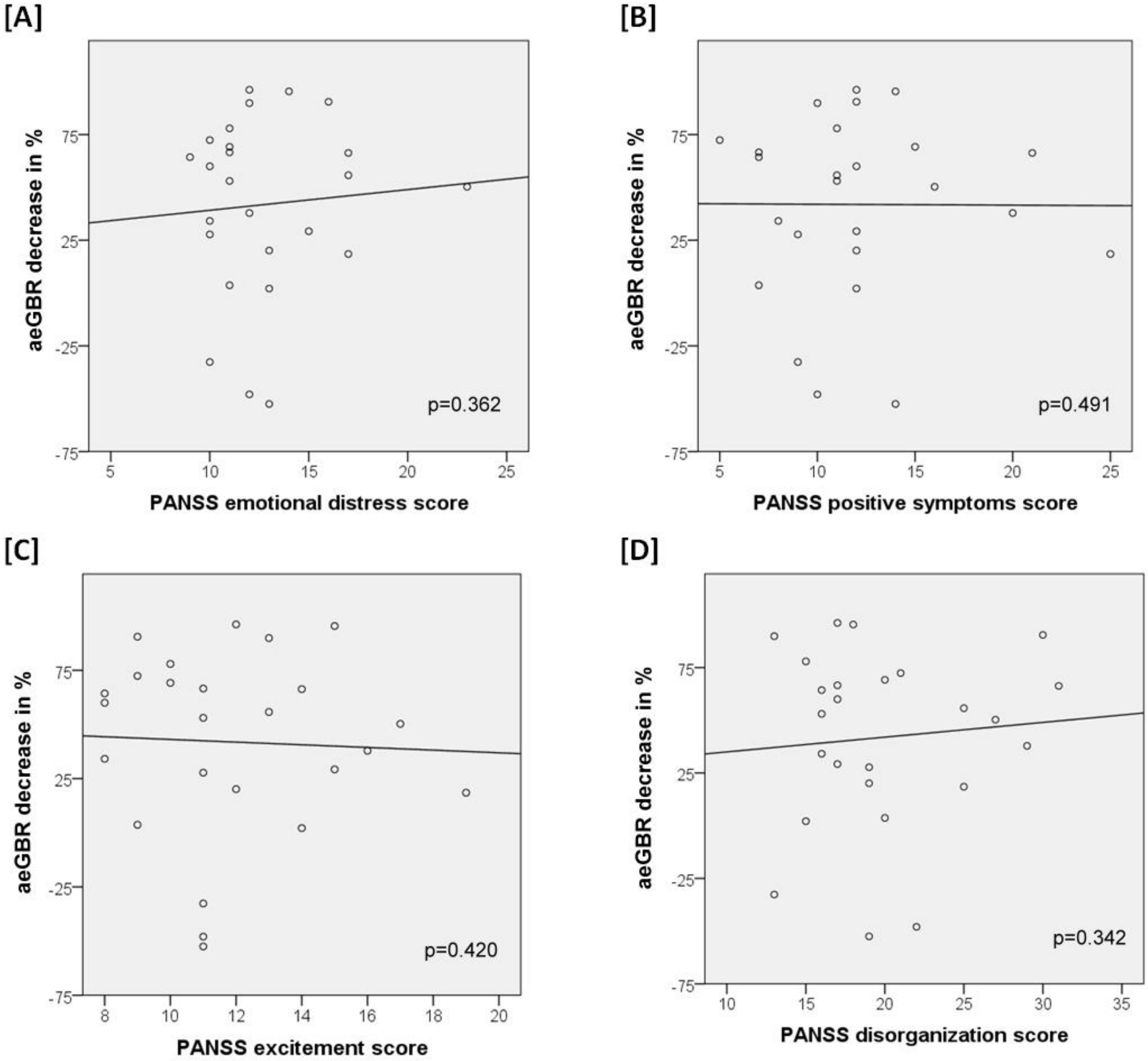


Fig.S1: Correlations between scores of the Positive and Negative Syndrome Scale (PANSS) factors and the relative decrease of aeGBR power: **[A]** Emotional distress factor (Pearson's r= 0.074), **[B]** positive symptoms factor (Pearson's r= -0.005), **[C]** excitement factor (Pearson's r= -0.043), and **[D]** disorganization factor (Pearson's r=0.086).

For the calculation of the baseline-activity, the mean spectral power in the time-range between 400 and 50ms pre-stimulus was selected. Saccadic spike potential artifacts (SPs) in the gamma frequency range [3] were controlled by an additional “radial electro-oculogram channel” (REOG), that was derived following the procedure described by Keren [4]. Independent component analysis (ICA) was applied to identify and remove blinks, drifts and SPs based on their characteristic topographies, time courses and frequency distributions [5]. A Fast Fourier Transform algorithm was used for the computation of mean power in the frequency range between 35–45 Hz. A paired-sample t-test was conducted to compare the mean power at Cz between placebo and ketamine condition.

There was no significant difference between the baseline power at Cz under placebo (mean = 0.26 µV², SD = 0.08) compared to the ketamine condition (mean = 0.27 µV², SD = 0.10); t(22)= -0.076 p=0.94 (Fig.S2).

There were no significant correlations between the power at Cz and the measures of aeGBR (power, PLF) or psychopathological measures (PANSS- and 5D-ASC-scores).

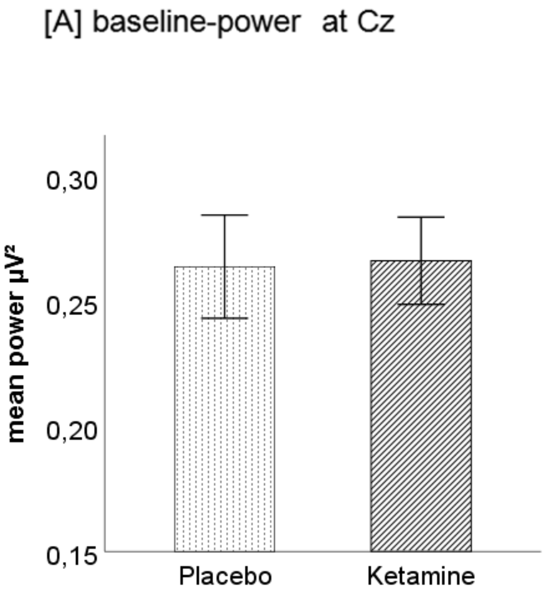


Fig.S2: mean baseline power at Cz 400 to 50ms prestimulus with error bars representing ± 1 standard errors of the mean.

**N100**

The same preprocessing of data was applied for the ERP-calculation. Grandaverages were computed for each subject. N100 was scored as the largest negativity in the interval 70–150ms. A paired-sample t-test was conducted to compare the mean N100 amplitude between placebo and ketamine condition.

The N100 amplitudes under ketamine (mean = -4.8 µV, SD = 2.3) were significantly reduced compared to the placebo condition (mean = -5.5 µV, SD = 2.6); t(24)= -2.117 p=0.045 (Fig.S3). There was no significant difference between N100 latency in the placebo (mean = 127 ms, SD = 15) and in the ketamine (mean = 124 ms, SD = 13) condition; t(24)= 1.283 p=0.212.

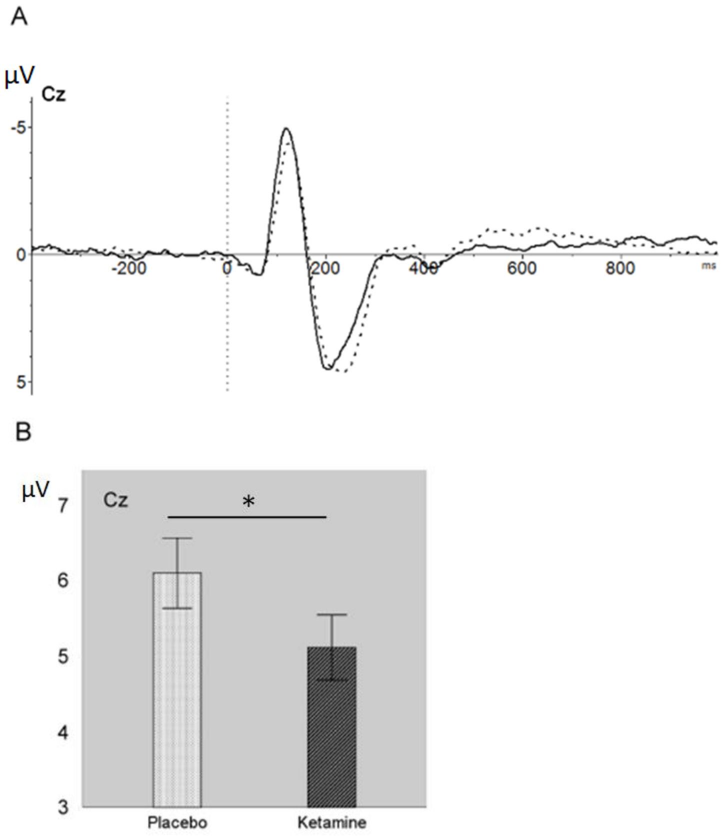


Fig.S3: **(A)** ERP elicited by a tone at Cz; grand average data (n=25) under ketamine (dashed line) compared to placebo; **(B)** mean N100 amplitudes with error bars representing ± 1 standard errors of the mean (* p<0.05).

## REFERENCES

1. de la Salle S, Choueiry J, Shah D, Bowers H, McIntosh J, Ilivitsky V, et al. Effects of Ketamine on Resting-State EEG Activity and Their Relationship to Perceptual/Dissociative Symptoms in Healthy Humans. Front Pharmacol. 2016;7:348.
2. Gandal MJ, Edgar JC, Klook K, Siegel SJ. Gamma synchrony: towards a translational biomarker for the treatment-resistant symptoms of schizophrenia. Neuropharmacology. 2012;62:1504-18.
3. Yuval-Greenberg S, Tomer O, Keren AS, Nelken I, Deouell LY. Transient induced gamma-band response in EEG as a manifestation of miniature saccades. Neuron. 2008;58:429-41.
4. Keren AS, Yuval-Greenberg S, Deouell LY. Saccadic spike potentials in gamma-band EEG: characterization, detection and suppression. Neuroimage. 2010;49:2248-63.
5. Carl C, Acik A, Konig P, Engel AK, Hipp JF. The saccadic spike artifact in MEG. NeuroImage. 2012;59:1657-67.
